# Robust deep learning‐based patient‐specific quality assurance prediction models for novel dual‐layer MLC linac

**DOI:** 10.1002/acm2.70286

**Published:** 2025-10-10

**Authors:** Qizhen Zhu, Xiaoyang Zeng, Zhiqun Wang, Heling Zhu, Yongguang Liang, Awais Ahmed, Bo Yang, Jie Qiu

**Affiliations:** ^1^ Department of Radiation Oncology Peking Union Medical College Hospital Chinese Academy of Medical Sciences and Peking Union Medical College Beijing China; ^2^ School of Computer Science and Engineering University of Electronic Science and Technology of China Chengdu Sichuan China; ^3^ School of Computer Science China West Normal University Nanchong Sichuan China

**Keywords:** deep learning, gamma passing rate, Halcyon linac, IMRT, prediction model, PSQA

## Abstract

**Purpose:**

This study investigates the feasibility of utilizing deep learning models to robustly predict patient‐specific quality assurance (PSQA) outcomes in fixed field intensity‐modulated radiation therapy (FF‐IMRT) plans on the Halcyon linear accelerator equipped with a novel dual‐layer multi‐leaf collimator (MLC). The study explores the integration of Shuffle Attention (SA) mechanisms and deep imbalance regression techniques to enhance the precision and robustness of deep learning‐based PSQA predictions. It ensures relative prediction robustness in the extreme imbalance distribution of gamma passing rate (GPR) values.

**Methods:**

Data from 214 FF‐IMRT treatment plans covering various treatment sites comprising 1394 beam orientations and corresponding Portal Dosimetry verification data were collected. Fluence maps calculated for each beam orientation served as inputs for the ResNet model. First, the SA module was introduced to improve the prediction accuracy of ResNet, resulting in the proposed Att‐ResNet model. Furthermore, to ensure prediction robustness in the GPR values with extreme imbalance distribution, we incorporated the Label Distribution Smoothing (LDS) technique, ultimately forming the ALDS‐ResNet method.

**Results:**

ALDS‐ResNet exhibited smaller mean absolute error (MAE) values than ResNet across all gamma criteria (1%/1 mm: 2.035 vs. 1.824, 2%/2 mm: 1.416 vs. 1.178, 3%/3 mm: 0.951 vs. 0.787). ALDS‐ResNet also demonstrated lower MAE values than ResNet for complex but important plan samples (GPR < 85, 1%/1 mm: 10.163 vs. 4.985, 2%/2 mm: 7.443 vs. 3.272, 3%/3 mm: 5.031 vs. 2.940). Compared to ResNet, ALDS‐ResNet achieved higher Pearson correlation coefficient (CC) values at 2%/2 mm and 3%/3 mm gamma criteria, measuring 0.7864 and 0.7852, respectively.

**Conclusions:**

The deep learning model based on ResNet shows promise for predicting GPR values in linacs with dual‐layer MLC. Integrating attention mechanisms with deep learning networks enhances the accuracy of PSQA predictions. The LDS technique is attributed to the substantial improvement in failed plan GPR prediction accuracy and robustness. Specifically, the deep learning model tailored for dual‐layer MLC linacs can be an auxiliary tool for physicists in identifying PSQA failure plans.

## 1 INTRODUCTION

With the rapid development of radiation therapy techniques, intensity‐modulated radiation therapy (IMRT) has been widely used in clinical treatment, which provides highly conformal dose coverage to the target volume and minimizes irradiation to the surrounding organs at risk (OARs). However, when compared to traditional 3‐dimensional conformal radiation therapy, the IMRT technique increases the complexity of treatment plans which can add to the dosimetric uncertainty during planning and delivery.^1^ Hence, prior to implementation, IMRT must be thoroughly verified for safety and reliability.^2^ Patient‐specific quality assurance (PSQA) is a vital process in the radiation therapy workflow and is recommended by TG119 and TG218 reports.^3^, ^4^ Before treatment delivery, PSQA is often performed by comparing the measured and calculated dose distributions using specific commercial detector arrays. However, the considerable workload and time‐consuming process are tough problems, and traditional measurement‐based PSQA methods have become increasingly unfeasible for busy centers.^5^ As a result, radiation oncology centers demand a more simplified, resource‐constrained PSQA approach for treatment plan verification.

In recent years, the applications of artificial intelligence techniques in radiotherapy have increased rapidly, including error detection and quality assurance (QA).^6^ Several studies have been conducted to develop prediction models based on conventional machine learning,^7^, ^8^ and deep learning techniques^9^, ^10^ to predict PSQA results, such as gamma passing rate (GPR), and use these to improve efficiency. By using PSQA prediction models, medical physicists can be informed which treatment plan has a larger possibility of QA failure before actual measurement and assist physicists in focusing on the PSQA for the few plans that are likely to fail. At the very beginning, various studies attempted to develop machine learning models by extracting the parameters related to the treatment plan uncertainty as model inputs, such as plan complexity metrics, linac performance parameters, and so on. Valdes et al.^11^, ^12^ made the first attempt to build a machine learning model for virtual PSQA using Poisson regression with Lasso regularization to predict the GPRs. Li et al.^13^ developed a classification model based on the Random Forest algorithm to predict whether treatment plans pass or fail QA. Lam et al.^14^ built prediction models using three different tree‐based machine learning algorithms (Adaboost, Random Forest, and XGBoost) to predict GPRs and achieved high prediction accuracy. However, conventional machine learning approaches rely heavily on the manual extraction of features and the process is laborious and may have an impact on the final performance. Hence, some studies investigated the performance of deep learning methods to predict PSQA outcomes. Interian et al.^15^ developed a convolutional neural network (CNN) and compared it against the Poisson regression model using the same patient QA data. The results showed that CNN had comparable accuracy compared with Poisson regression. Ono et al.^16^ compared the performance of three VMAT QA prediction models which used the artificial neural network (ANN), regression tree analysis, and multiple regression analysis separately. Their study reported that the ANN model had a smaller prediction error among the 3 models. Huang et al.^17^ built UNet++ based on UNet using the planar dose distribution as input to predict GPRs, QA classification results, and dose difference position. Though there are signs of progress in recent years, the potential of deep learning model development and training strategies for PSQA outcomes prediction has not been thoroughly investigated. Besides, previous studies mainly focused on ordinary single‐layer MLC linac. Recently, a novel O‐ring gantry linac ‘Halcyon’ equipped with a jawless stacked‐and‐staggered dual‐layer MLC system was released (Varian Medical Systems, Inc., Palo Alto, CA). The stacked‐and‐staggered configuration can achieve an effective 5.0 mm resolution at the isocenter (the MLC leaf width of each layer is 10 mm), allowing Halcyon to reduce inter‐leaf leakage and intra‐leaf transmission. Furthermore, because of the high leaf positioning accuracy, quick leaf motion speed, and low electron contamination, this jawless dual‐layer MLC device has many advantages for intensity‐modulated therapies.^18^

**FIGURE 1:**
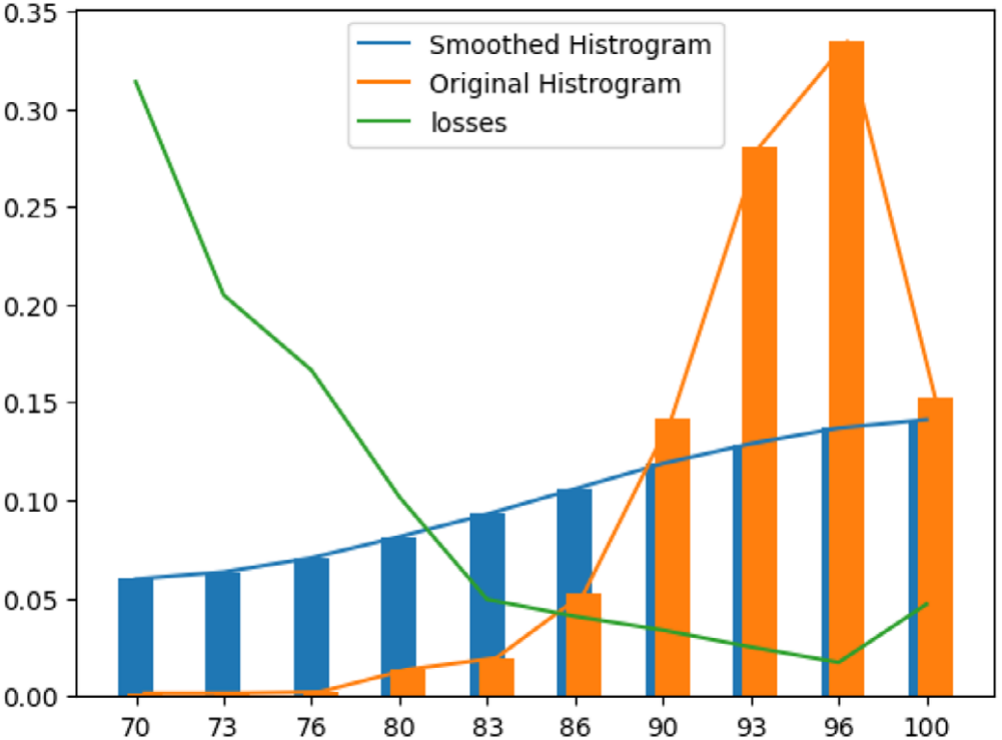
GPRs dataset imbalance and bias problem.

One significant issue in the GPRs prediction task is the PSQA data distribution extreme imbalance problem because, in clinical practice, most treatment plans can successfully pass, with only a few plans failing. In the algorithmic realm, the performance of machine learning and deep learning algorithms tends to decline significantly when dealing with unbalanced data, particularly when handling underrepresented samples. Figure 1 shows an introductory experiment about how deep learning performance varies with the data distribution; we plot the plan GPRs histogram and show the ResNet prediction result in different ranges. Mean Absolute Error (MAE) was used as the loss function on the validation set to quantify the difference between predicted and ground truth GPRs. In the histogram, the y‐axis represents the proportion of samples within each GPR range, while the green curve indicates the average validation loss (MAE); lower values correspond to better model performance. Notably, the height of the histogram is negatively correlated with the loss curve, suggesting that the model performs worse in GPR ranges with fewer training samples. This highlights the adverse effect of data imbalance on regression accuracy. Worse still, in such rare sample scenarios, the model tends to refer to information from typical samples to make predictions, leading to overestimation. Furthermore, the PSQA task presents asymmetric risks: predicting a successful plan as a failure merely requires redesigning it manually, whereas predicting a failed plan as successful poses potential safety risks and medical accidents, which could threaten patients' lives. A model that performs well only on the overall dataset is still insufficient for clinical application. Only a model that can robustly perform well on samples with safety risks (also challenging for deep learning) has the potential for clinical use.

Traditional work on this data imbalance problem has primarily focused on classification tasks, while GPRs prediction application is a regression problem. Most previous work on imbalanced regression has adapted the Synthetic Minority Over‐sampling Technique (SMOTE) algorithm to regression scenarios by directly interpolating inputs and targets or using Gaussian noise augmentation to create synthetic samples for predefined rare target regions.^19^, ^20^, ^21^ However, these methods have several drawbacks. First, the affected areas of the plans are different, and the interpolated plan data may need to be revised, which could lead to doubts about the model's reliability. Additionally, they need to consider the distance between targets and instead heuristically divide the dataset into rare and frequent sets, then apply classification‐based methods. Yuzhe Yang et al.^22^ systematically studied the problem of imbalanced regression and showed that when encountering rare patterns, the deep learning model tends to follow information from the common patterns, leading to inconsistent predictions. On the other hand, in regression problems, samples with similar label values usually exhibit certain pattern similarities. Therefore, Label Distribution Smoothing (LDS) was proposed to use kernel density estimation to learn the effective imbalance in datasets with continuous targets.

In this study, we developed a deep learning network, ALDS‐ResNet, which integrates with the attention module and deep imbalance regression module LDS to robustly predict GPRs for Halcyon linac with a dual‐layer MLC system. The predictions were made for fixed field‐IMRT (FF‐IMRT) plans of various treatment sites, including head and neck (H&N), chest, abdomen, and pelvis. Attention mechanisms can enable a neural network to focus on all the relevant elements of the input precisely, which has become a key component for improving deep neural networks’ performance. Therefore, we introduced the Shuffle Attention (SA) module^23^ into ResNet^24^ and the LDS module to improve robustness and the performance of PSQA outcomes prediction for Halcyon linac. This study aimed to firstly investigate the feasibility of building a deep learning model in predicting the GPRs at different gamma criteria for Halcyon linac; secondly, compare the performance differences of the deep learning models with and without the SA module and examine whether the attention mechanism can improve the model performance; and finally, to assess whether the LDS module can ensure the robustness to PSQA highly imbalanced GPRs data distribution.

## 2 METHODS AND MATERIALS

### 2.1 Data collection

214 FF‐IMRT treatment plans (containing 1394 beam fields) for various treatment sites were collected retrospectively from December 2020 to July 2021. There were 19 head and neck plans, 142 chest plans, 31 abdominal plans, and 22 pelvic plans among these. All plans were used the sliding window technique and generated based on Eclipse TPS version 15.6 and delivered by Halcyon 2.0 linac equipped with SX2 dual‐layer MLC (Varian Medical System, Palo Alto, CA). The dose distribution was calculated using the anisotropic analytic algorithm (AAA, ver. 15.6.06, Varian Medical Systems, Palo Alto, CA) with a dose calculation grid of 2.5 mm, and the plan optimization algorithm was photon optimization (PO, ver. 15.6.06, Varian Medical Systems, Palo Alto, CA) algorithm. According to the approach recommended in the TG‐218 report,^4^ PSQA measurements were performed before treatment delivery using actual angles for each beam by Portal Dosimetry. Dose calibration was performed every day before data collection. All gamma analyses were performed with 1%/1 mm, 2%/ 2 mm, and 3%/2 mm criteria at 10% threshold of the maximum dose (only points with doses greater than 10% of the global maximum dose per beam were analyzed). Absolute dose mode and global normalization were used for gamma analyses. We exported the calculated fluence maps from TPS in DICOM format and used them as network input.

### 2.2 Data preprocessing

Raw fluence maps of 1394 beam fields have varied spatial resolutions and sizes; several pre‐processing steps were performed. The 2D fluence maps were resampled to 1 mm × 1 mm spatial resolution and then cropped to 400 × 340 pixels to remove the redundant background. Finally, before being fed into the network, the pixel values of the input images were rescaled to [0,1] by Min‐Max normalization.

### 2.3 Network architecture

**FIGURE 2:**
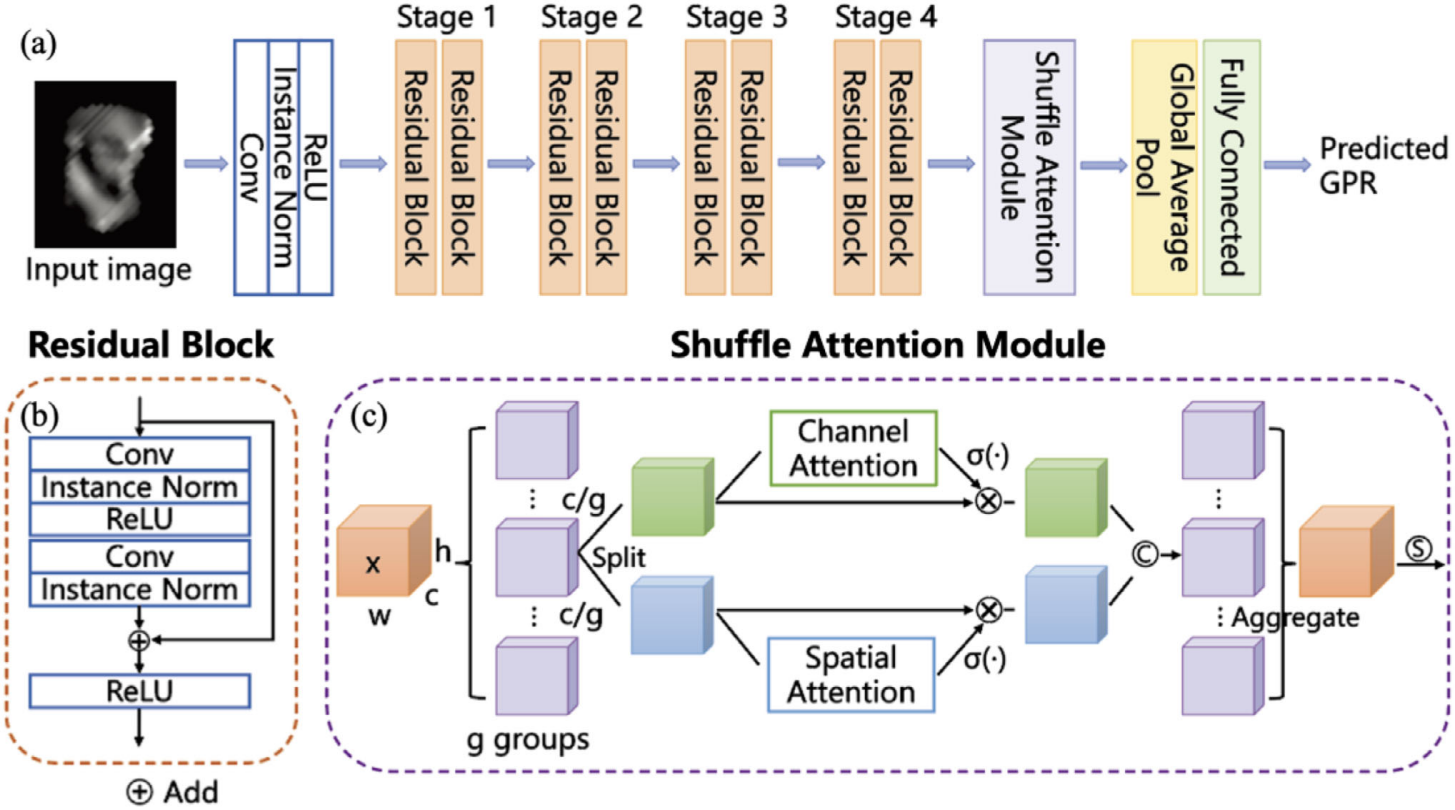
The architecture of the shuffle attention‐based ResNet in this study. (a) Overall architecture of the network, (b) Residual block, (c) Shuffle attention module.

Figure 2 illustrates the overall architecture and basic components of our proposed network. ResNet^24^ is a classical and efficient structure and has been widely used in many medical image tasks.^25^, ^26^ When a neural network reaches a certain depth, increasing the number of layers cannot bring improvements due to the gradient vanishing and network degradation. ResNet proposed the idea of residual learning to alleviate the above problems by fitting a residual function (wherein the input and output of the residual block are, respectively). Here, we employed ResNet18 as the feature extraction backbone in our network, which mainly consists of four stages, each including two cascaded Residual Blocks. The Residual Blocks comprise Convolution, Instance Normalization, and ReLU activation layers to learn the residual mapping.

SA^23^ module is incorporated into the network behind the last Residual Block. The SA module combines spatial and channel attention mechanisms to apply pixel‐wise and channel‐wise weights to the feature maps, facilitating the network to focus on important information while suppressing irrelevant regions. In the SA module, the feature maps are divided into groups and then fed into channel attention and the spatial attention layer in parallel, which is implemented by a lightweight SE attention module^27^ and Group Norm operation, respectively. Then, all feature groups are concatenated, and a “Channel Shuffle” operator is utilized to promote the information interaction between different channels. The outputs of the SA module are fused into a one‐dimensional vector through a Global Average Pool (GAP) layer. Finally, a fully connected layer outputs the gamma passing rates (GPRs).

### 2.4 Solving the deep imbalance problem

Due to the prevalence of treatment plans with high GPRs in clinical practice, a significant data imbalance arises, leading the model to demonstrate a strong bias toward overpredicting GPRs. However, the critical aspect lies in prioritizing attention to cases with low passing rates, as they could indicate patient risks or medical incidents.

LDS^22^ shows that deep learning models tend to follow prediction information from common patterns when encountering rare patterns, leading to inconsistent predictions. On the other hand, in regression problems, samples with similar label values usually exhibit certain pattern similarities. Therefore, a method was proposed to convolve the label distribution with a symmetric kernel and empirical density distribution. The kernel density estimation of the label distribution is obtained by extracting the kernel‐smoothed version, and the smoothed distribution is then used for reweighting.

As shown in Equation (1), we applied Gaussian smooth kernel reweighting to adjust the loss function using a continuous probability density function. This smoothness allowed us to effectively handle the skewed distribution of GPR values and prioritize accurate predictions for low GPR instances. During training, the smooth kernel weights were computed based on the distribution of GPR values in the training dataset, and the corresponding loss function is defined in Equation (2). The model was encouraged to focus on low GPR values by reweighting the loss function using the smooth kernel. This resulted in a more robust and reliable PSQA prediction framework.

(1) $$\bar{p}\left(y\right)=\int \frac{1}{\sqrt{2\pi }\sigma }\exp\left(-\frac{{\left(y-y^{\prime }\right)}^{2}}{2\sigma^{2}}\right)\cdot p\left(y^{\prime }\right)dy^{\prime }$$

(2) $$\begin{array}{cc} L_{GPRs} & =Weighted\_MAE(y,\hat{y})\\ & =\frac{1}{N}\sum_{i=1}^{N}\frac{\bar{p}\left(y_{i}\right)}{p(y_{i})}\left|y_{i}-{\hat{y}}_{i}\right| \end{array}$$

The σ is a hyperparameter optimized to 1 in the final experiment. y denotes the GPR label values,  p(y) represents the original GPR distribution, and *p* (*y*) indicates the smoothed distribution. We first apply the Gaussian kernel smooth to estimate the effective density of the GPR label values and then perform importance sampling^28^ to get the final reweight loss form. Figure 1 also presents a smoothed GPR distribution as an example.

By training the model with smoothed weights, the feature representations allowed the model to capture meaningful patterns and relationships between the input features and the PSQA outcomes of the underrepresented samples. Consequently, this approach enhanced the model's ability to handle data imbalance and effectively leverage information from cases with low gamma passing rates.

### 2.5 Model training strategy and evaluation

We used the 5‐fold cross validation^29^ for evaluation. The ratio of the training, validation, and test sets was 7:1:2. The models were trained with mean absolute error loss using the Adam optimizer for 100 epochs. The learning rate for 1%/1 mm, 3%/2 mm, and 2%/2 mm were set as 3e‐4. The batch size was set as 16. We used Pytorch Library to implement our network. Model training costs 8.7 GB of memory and 30 h on an NVIDIA Tesla A100 GPU. Models that achieved the lowest loss on the validation set were used for testing, and all evaluation metrics were reported as the average value in cross‐validation. We calculated the mean absolute error (MAE), root mean squared error (RMSE), and Pearson correlation coefficient (CC) between the predicted and measured GPRs to evaluate our models. CC > 0.7 is a high correlation; 0.4 < CC < 0.7 is a moderate correlation; CC ≤ 0.4 is a low correlation.

## 3 RESULTS

### 3.1 Data distribution

**FIGURE 3:**
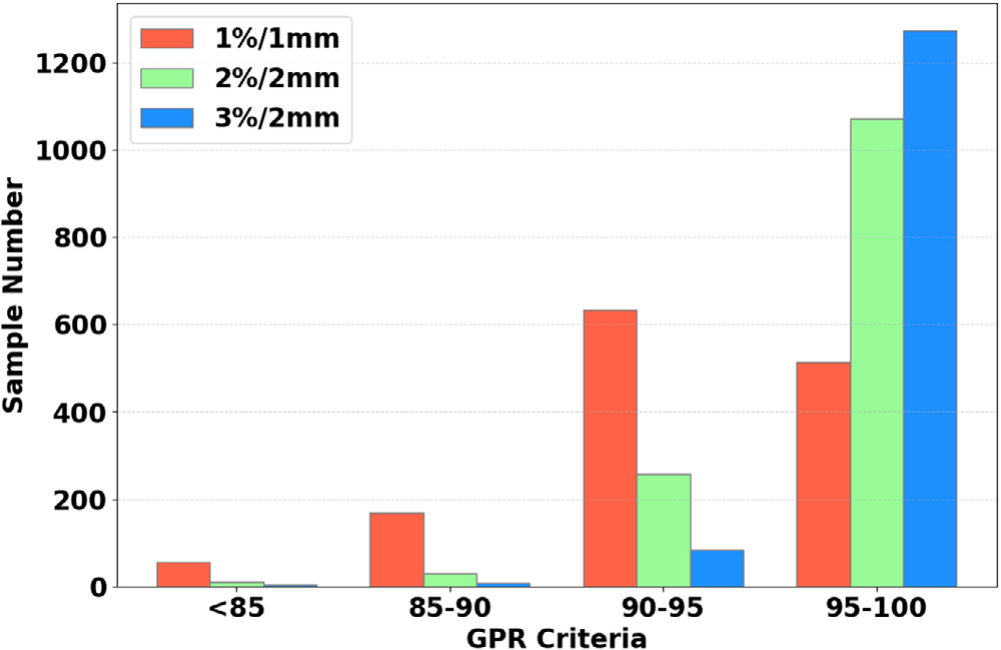
GPRs distribution in all criteria.

The distributions of measured GPRs of FF‐IMRT plans are shown in Figure 3. This dataset consists of a total of 1370 beam fields. Among them, 96 (6.9%) beam fields had GPRs lower than 95% at 3%/2 mm gamma criteria, 36 (2.8%) beam fields had GPRs lower than 90% at 2%/2 mm gamma criteria, and 229 (16.4%) beam fields had GPRs lower than 90% at 1 mm/1% gamma criteria.

### 3.2 GPRs prediction accuracy

Table 1 and Table 2 show the MAE and RMSE of prediction results for test sets under different gamma criteria in ResNet, Att‐ResNet and ALDS‐ResNet, respectively. To further demonstrate the robustness against imbalanced GPRs distributions, we present the overall performance on the test set and separately show the performance for GPRs ranges of < 85%, 85%–90%, 90%–95%, and 95%–100%. The primary focus of this work, and where the most significant performance improvement is observed, is within the 0%–85% range in ALDS‐ResNet.

**TABLE 1:** MAE for GPRs of ResNet, Att‐ResNet, and ALDS‐ResNet.

	1%/1 mm			2%/2 mm			3%/2 mm		
GPR range	ResNet	Att‐ResNet	ALDS‐ResNet	ResNet	Att‐ResNet	ALDS‐ResNet	ResNet	Att‐ResNet	ALDS‐ResNet
95–100	1.760	1.211	1.375	1.007	0.820	0.785	0.476	0.665	0.516
90–95	1.169	1.422	1.552	0.742	0.781	0.982	0.590	0.560	0.607
85–90	3.500	2.333	2.171	2.628	1.709	1.803	2.012	1.251	1.369
<85	10.163	6.671	4.985	7.443	4.709	3.272	5.031	3.519	2.940
**All**	**2.035**	**1.722**	**1.824**	**1.416**	**1.110**	**1.178**	**0.951**	**0.835**	**0.787**

**TABLE 2:** RMSE for GPRs of ResNet, Att‐ResNet, and ALDS‐ResNet.

	1%/1 mm			2%/2 mm			3%/2 mm		
GPR range	ResNet	Att‐ResNet	ALDS‐ResNet	ResNet	Att‐ResNet	ALDS‐ResNet	ResNet	Att‐ResNet	ALDS‐ResNet
95–100	2.288	1.898	2.163	1.257	1.162	1.175	0.636	0.855	0.759
90–95	1.529	1.912	2.057	0.960	1.223	0.982	0.802	0.779	0.829
85–90	3.734	2.913	2.600	2.906	2.353	2.673	2.317	1.760	1.740
<85	11.062	8.217	6.455	8.333	6.065	4.428	5.927	4.724	4.012
**All**	**3.298**	**2.727**	**2.698**	**2.354**	**1.882**	**1.898**	**1.697**	**1.428**	**1.350**

**FIGURE 4:**
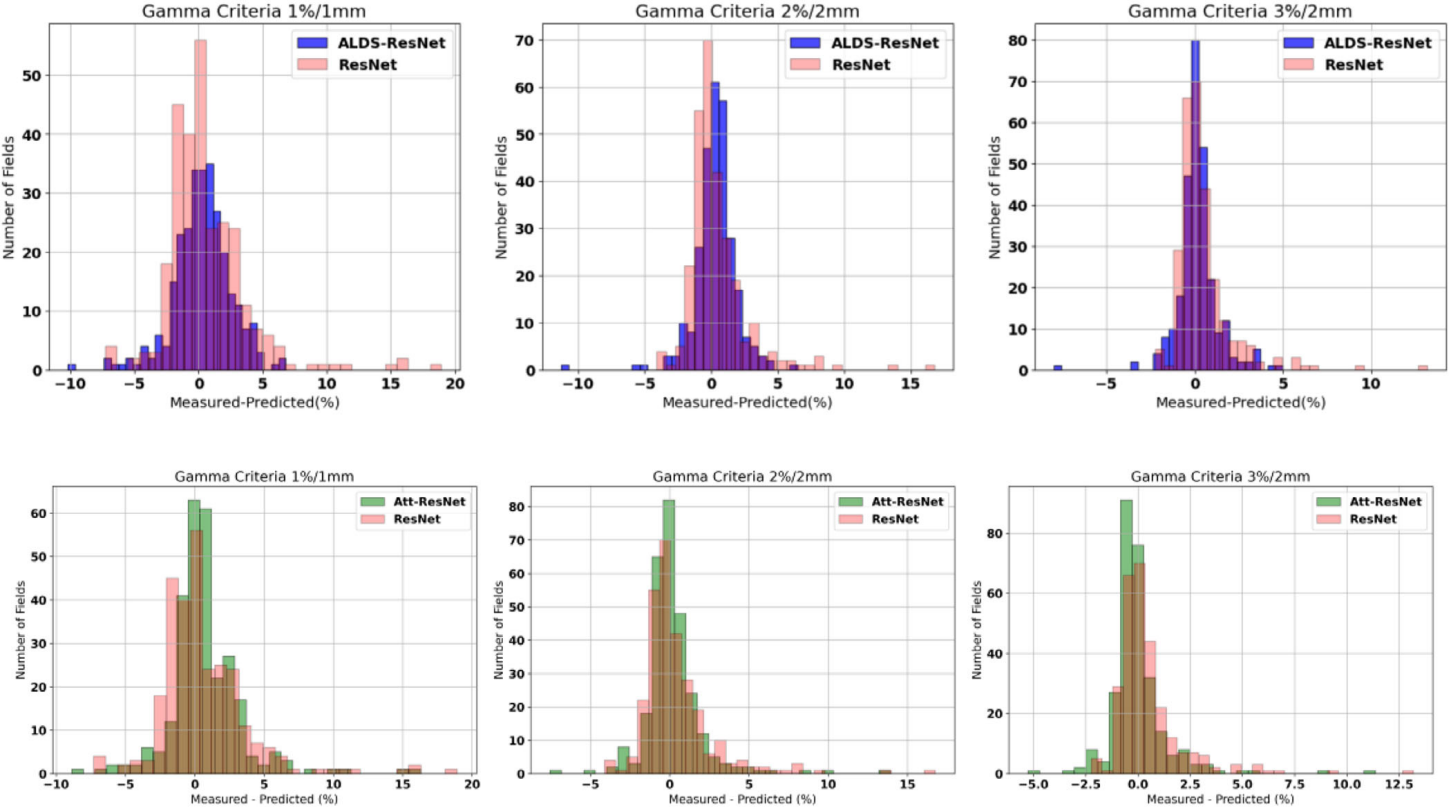
The error histograms of the ResNet, Att‐ResNet, and ALDS‐ResNet.

**FIGURE 5:**
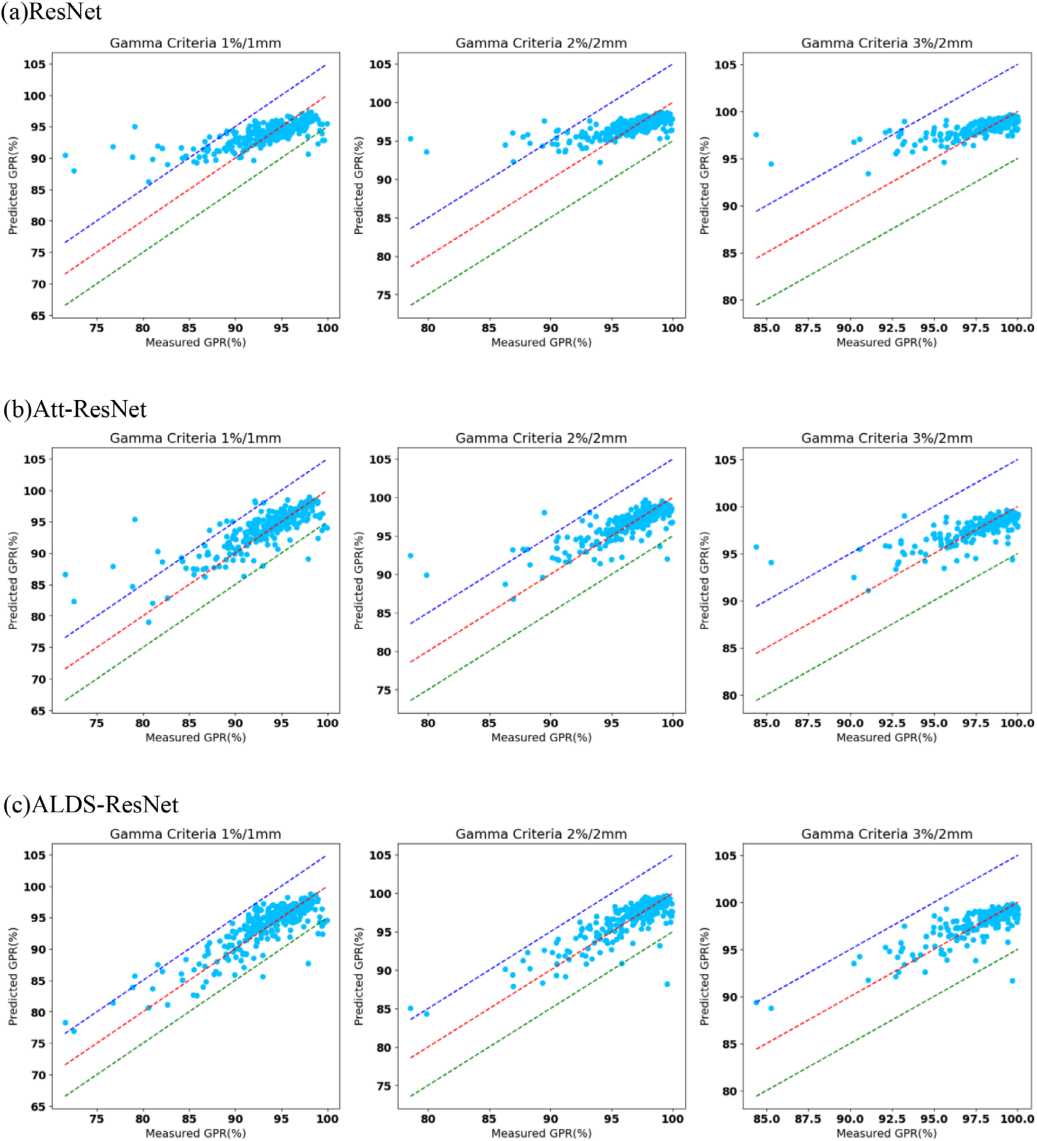
Predicted vs. Measured GPRs under three different gamma criteria in various models, with the red line representing a perfect prediction and the blue and green line representing a 5% error margin from measurements.

Figure 4 shows a better visualization of the predicted value at all gamma criteria from ResNet, Att‐ResNet, and ALDS‐ResNet, in which the predicted GPRs are plotted against the actual measured GPRs. To analyze the prediction results in detail, Figure 5 scatter plots the predicted GPRs and actual measured GPRs using the different gamma criteria from ResNet, Att‐ResNet, and ALDS‐ResNet. The red line represents a perfect prediction, and the blue and green lines represent a 5% error margin from measurements. We performed a Pearson correlation test to evaluate the correlation between predicted GPRs and actual measured GPRs, as depicted in Table 3. The CC of ResNet, Att‐ResNet, and ALDS‐ResNet presented highly positive correlations, with Att‐ResNet and ALDS236 ResNet showing larger CC at 2%/2 mm and 3%/2 mm gamma criteria.

**TABLE 3:** Pearson correlation coefficient (CC) of predicted GPRs and measured GPRs of ResNet, Att‐ResNet, and ALDS‐ResNet.

	CC (*p*‐value)		
	ResNet	Att‐ResNet	ALDS‐ResNet
1%1 mm	0.7489 (*p*<0.01)	0.8014 (*p*<0.01)	0.7939 (*p*<0.01)
2%/2 mm	0.7285 (*p*<0.01)	0.7911 (*p*<0.01)	0.7864 (*p*<0.01)
3%/2 mm	0.7269 (*p*<0.01)	0.7636 (*p*<0.01)	0.7852 (*p*<0.01)

## 4 DISCUSSION

The main benefit of the PSQA prediction model is that it can alert clinical physicists to which treatment plans may not pass QA measurements and help them focus on the small portion of failed QA treatment plans. This can reduce workload and improve workflow efficiency. Previous studies have shown that it is feasible to establish PSQA prediction models using deep learning techniques. However, deep learning is susceptible to imbalanced data distribution, and its performance decreases in underrepresented data. In clinical practice, failed plan samples are rare but more important. Current research lacks robustness studies on imbalanced distributions, and most related algorithmic studies focus on classification problems, lacking studies on imbalanced regression. On the other hand, there is still room for improvement in prediction accuracy in current studies. To our knowledge, there is no deep learning model for the Halcyon linac equipped with a novel dual‐layer MLC system.

Tables 1 and 2 numerically demonstrate the efficacy of the SA and LDS modules. All models' performance significantly dropped in samples with GPRs below 85. The ResNet model MAE is 10.163 for these underrepresented samples under the 1%/1 mm gamma criteria, strikingly different from the MAE of 1.760 for 95–100 GPR samples. All gamma criteria and models show this pattern, demonstrating deep learning algorithms' problems with skewed data distributions that underrepresent samples.

The attention mechanism enhances ResNet's performance. Att‐ResNet's MAE in the 95–100 GPR range for the 1%/1 mm gamma criterion is 1.211, compared to ResNet's 1.760, indicating a significant error reduction. This pattern occurs across all GPR ranges and gamma criteria. Under the 1%/1 mm criteria, Att‐ResNet has a superior overall MAE of 1.722 than ResNet's 2.035. These results show that the attention mechanism helps the model focus on more informative features, improving prediction accuracy. Table 3 also showcases the improvement of the attention mechanism from the correlation view.

The LDS module considerably improves underrepresented samples with GPRs below 85. Under the 1%/1 mm gamma requirements, the ALDS‐ResNet model has an MAE of 4.9853, significantly better than ResNet's 10.163 and Att‐ResNet's 6.6705. The LDS module handles imbalanced data distributions well, as seen by this improvement across all gamma criteria. The LDS module smoothes label distributions to help the model generalize to underrepresented samples, improving performance where needed. The LDS module improves performance on underrepresented samples but increases mistakes in representative regions of the 95–100 GPR range. Figure 5 further supports the effectiveness of LDS in rare cases: significantly fewer than 5% of ALDS‐ResNet's predictions for GPR < 90 exceed a 5% error, compared to those of Att‐ResNet. This confirms LDS's strength in handling rare but clinically significant samples. This situation is acceptable since, in clinical practice, underestimation usually poses no safety risks.

Figure 4 compares the error distribution between the traditional ResNet, Att‐ResNet, and ALDS‐ResNet. Att‐ResNet demonstrates comprehensively superior performance compared to ResNet. Compared with ALDS‐ResNet, the conventional ResNet has more predictions with low errors but also a much higher number of significant errors. Combined with Figure 5 and the previous tables, it is evident that these significant errors mostly come from underrepresented but safety‐critical samples (false positives). The substantial number of low‐error samples is mainly due to good performance in the 95–100 GPR range. Figure 5 provides a more detailed illustration showing that as the selected GPR decreases, the false positive increases significantly, leading to a decline in performance. Additionally, it demonstrates how the LDS module for imbalanced regression mitigates this performance decline in the low GPR area. Although ALDS‐ResNet slightly sacrifices performance for samples with GPRs between 95–100, these are primarily false negatives that pose no safety risks.

IMRT, involving modulation of extensive machine parameters, is a complex technology, and there are many sources of errors or uncertainties in IMRT planning and delivery processes. Therefore, PSQA prior to treatment delivery is necessary to ensure treatment reliability and safety.^4^ In the beginning, researchers used manually designed features (plan complexity metrics and machine delivery parameters) as inputs to build PSQA prediction models through conventional machine learning algorithms.^5^ However, the step of manually extracting features is a labor‐intensive and time‐consuming process. Compared to conventional machine learning approaches, the use of deep learning approaches has certain advantages: Firstly, with input data increased, the conventional machine learning models will be saturated and new features have to be designed to improve performance.^15^, ^30^ Secondly, through transfer learning techniques, a well‐trained deep learning model can be applied to other practical situations without training a new model from scratch. From this point of view, it will make PSQA prediction models widely used in real‐world clinical practice.

The FF‐IMRT plans we used for developing the deep learning models were from four different treatment sites. As a result, our study evaluates the performance of deep learning models more comprehensively for PSQA prediction tasks of dual‐layer MLC linac. Our results show that the MAE and RMSE for our models' measured and predictive GPRs rose as the gamma criteria got stricter. This is consistent with our perceptions of the prediction of GPRs at different gamma criteria. The measured GPRs themselves have an impact on the models' accuracy. During the model training process, the samples with high measured GPRs account for a large proportion, so the prediction results of higher GPRs are more accurate, which has also been seen in the previous studies.^13^, ^14^, ^17^ With gamma criteria becoming stricter, the distribution of measured GPRs of training samples is more heterogeneous, which could be the main reason for the above phenomenon. The SA mechanism and ResNet combination, which we called Att‐ResNet in this study, showed an improved prediction accuracy. The main improvements appeared in the MAE between measured GPRs and predictive GPRs. At 1%/1 mm gamma criteria and a GPR range less than 85, the ALDS‐ResNet achieved a 5.177% improvement over the conventional ResNet. The results of our study indicate that the SA mechanism integrated with LDS has the potential to improve the performance and robustness of the deep learning model in such prediction tasks.

Halycon was released in 2017 with a revolutionary dual‐layer MLC. This innovative design aims to reduce leakage between MLC leaves and achieve quick beam modulation. The faster MLC leaves motion and gantry rotation speed and automatical couch positioning of Halcyon allow a more efficient workflow for radiotherapy.^18^, ^31^ Based on this, Varian's online adaption treatment platform Ethos(Varian Medical Systems, Inc., Palo Alto, CA, USA) uses the same dual‐layer MLC configuration and O‐ring gantry design. For online adaption radiotherapy, the PSQA program needs to be completed within a short time to confirm the safety of treatment plans, and consequently, a more efficient and more streamlined PSQA mode is indispensable.^32^ Considering the potential of the dual‐layer MLC system, specific PSQA prediction models based on deep learning methods will be a useful auxiliary tool for improving work efficiency and optimizing the whole workflow. However, it is essential to highlight that using prediction models can't replace measurement‐based QA at this stage; instead, it can be used as an auxiliary tool to a conventional measurement‐based program. In this work, we only concentrated on predicting GPRs measured by Portal Dosimetry (2D dose distribution), but similar results can be expected for other measurement practices for 3D dose distribution. The limitations of our study should be noted. In our research, deep learning models were developed using treatment plans from only one line and one center. Due to different PSQA practice methods and dosimetric linac modeling, the models developed at our center might not apply to another center.

## 5 CONCLUSION

For Linac, with a novel dual‐layer MLC configuration, the deep learning model based on ResNet shows the potential to predict the GPRs' value. Combining LDS and attention mechanisms with deep learning networks can improve the robustness and accuracy of PSQA prediction tasks. The specific deep learning models for dual‐layer MLC linac can help clinical physicists identify plans’ quality more efficiently.

## AUTHOR CONTRIBUTIONS

Qizhen Zhu, and Xiaoyang Zeng investigated the concept. Qizhen Zhu, Xiaoyang Zeng, Zhiqun Wang, Yongguang Liang, and Heling Zhu performed the experiments. Qizhen Zhu and Xiaoyang Zeng performed the statistical analysis. Awais Ahmed provided suggestions for model training and evaluation strategies All authors edited the manuscript. Bo Yang and Jie Qiu are guarantors of the integrity of the entire study. All authors reviewed and approved the final manuscript.

## CONFLICT OF INTEREST STATEMENT

The authors declare no conflicts of interest.

## Data Availability

The raw data supporting the conclusions of this article will be made available by the corresponding authors, without undue reservation.
